# Prediction of Clinical Remission with Adalimumab Therapy in Patients with Ulcerative Colitis by Fourier Transform–Infrared Spectroscopy Coupled with Machine Learning Algorithms

**DOI:** 10.3390/metabo14010002

**Published:** 2023-12-19

**Authors:** Seok-Young Kim, Seung Yong Shin, Maham Saeed, Ji Eun Ryu, Jung-Seop Kim, Junyoung Ahn, Youngmi Jung, Jung Min Moon, Chang Hwan Choi, Hyung-Kyoon Choi

**Affiliations:** 1 College of Pharmacy, Chung-Ang University, Seoul 06974, Republic of Korea; dud612@cau.ac.kr (S.-Y.K.); mahamsaeed@cau.ac.kr (M.S.); jeryu1029@cau.ac.kr (J.E.R.); rara0630@cau.ac.kr (J.-S.K.); wnsdud96@cau.ac.kr (J.A.); xhfn99@cau.ac.kr (Y.J.); 2 Department of Internal Medicine, College of Medicine, Chung-Ang University, Seoul 06973, Republic of Korea; mdthepage@cauhs.or.kr (S.Y.S.); jmmoon@cau.ac.kr (J.M.M.)

**Keywords:** prediction, adalimumab, ulcerative colitis, Fourier transform–infrared spectroscopy, machine learning algorithms

## Abstract

We aimed to develop prediction models for clinical remission associated with adalimumab treatment in patients with ulcerative colitis (UC) using Fourier transform–infrared (FT–IR) spectroscopy coupled with machine learning (ML) algorithms. This prospective, observational, multicenter study enrolled 62 UC patients and 30 healthy controls. The patients were treated with adalimumab for 56 weeks, and clinical remission was evaluated using the Mayo score. Baseline fecal samples were collected and analyzed using FT–IR spectroscopy. Various data preprocessing methods were applied, and prediction models were established by 10-fold cross-validation using various ML methods. Orthogonal partial least squares–discriminant analysis (OPLS–DA) showed a clear separation of healthy controls and UC patients, applying area normalization and Pareto scaling. OPLS–DA models predicting short- and long-term remission (8 and 56 weeks) yielded area-under-the-curve values of 0.76 and 0.75, respectively. Logistic regression and a nonlinear support vector machine were selected as the best prediction models for short- and long-term remission, respectively (accuracy of 0.99). In external validation, prediction models for short-term (logistic regression) and long-term (decision tree) remission performed well, with accuracy values of 0.73 and 0.82, respectively. This was the first study to develop prediction models for clinical remission associated with adalimumab treatment in UC patients by fecal analysis using FT–IR spectroscopy coupled with ML algorithms. Logistic regression, nonlinear support vector machines, and decision tree were suggested as the optimal prediction models for remission, and these were noninvasive, simple, inexpensive, and fast analyses that could be applied to personalized treatments.

## 1. Introduction

Ulcerative colitis (UC), as an inflammatory bowel disease (IBD), is a chronic disease characterized by broad mucosal inflammation of the rectum and colon [1]. Over the past few years, the incidence of UC has been consistently increasing, especially in Asia, including South Korea [1].

The main goal of UC treatment is to improve the quality of life of patients by inducing and maintaining clinical remission [2]. Anti–tumor necrosis factor-alpha (anti-TNF-α) drugs, including infliximab, adalimumab, and golimumab, have been reported to show efficacy in inducing remission in patients with moderate to severe UC who are refractory to conventional drugs [3,4]. However, not all patients have successful treatment outcomes [3]. In clinical settings, not all patients treated with anti-TNF drugs can achieve clinical remission, symptom reduction, and improved quality of life. Some patients may not respond to the treatment at all, which is known as primary nonresponse (PNR) [5]. Additionally, even among those who initially respond to the treatment, some may experience a loss of response (LOR) over time and may not be able to maintain remission within a year [6]. The incidence of PNR to anti-TNF drugs has varied between clinical trials and clinical practice, with rates ranging from 10 to 20% and 13 to 30%, respectively. Despite the widespread use of these drugs, there is currently no consensus on the rate of LOR to TNF-α antagonists [7]. For example, some studies have reported that a dose increase after 12 weeks of therapy is required in 23–46% of patients, while others have reported that drug discontinuation occurs in 5–13% of patients [7]. The predictors of anti-TNF therapy outcomes may help in selecting patients who are likely to achieve and maintain clinical remission without wasting unnecessary medical resources and time and without resulting in adverse events.

The efficacy of anti-TNF therapy in patients with IBD can be predicted using a variety of factors from both clinical (gender, patient factors, age, weight, disease duration, phenotype, smoking status, and medical comorbidities) and experimental (immunological markers, genetic markers, microbiome analysis, endoscopic evaluation, and serological markers) perspectives using invasive or noninvasive biological samples [8]. This provides a comprehensive approach to optimizing responses to anti-TNF therapy and managing the disease effectively [8]. Several studies have explored the use of anti-TNF drugs for treating UC and have incorporated machine learning (ML) algorithms to analyze the data collected. This approach has important implications for clinical decision-making, as it has the potential to assist healthcare providers in determining which patients may benefit from the continued use of costly medications [9]. For example, a study using ML and data from the first 6 weeks of vedolizumab therapy for UC showed an accurate prediction of corticosteroid-free endoscopic remission after 52 weeks [10]. Also, the effect of azathioprine on mucosal healing in patients with IBD has been evaluated by employing artificial neural networks to predict mucosal remission [11]. Furthermore, as a tool to assist in therapeutic decisions, an ML model was developed to predict disease activity in UC patients treated with anti-TNF-α drugs [12]. ML-based analysis of gene expression and DNA methylation in blood samples of IBD patients was employed for early prediction of therapy response to anti-TNF (infliximab) treatment in IBD patients [13]. However, most such prediction strategies have limitations in their application in real clinical settings, and there is no single marker satisfying all criteria available as an optimal prognostic predictor [14]. However, the study platforms used to gather this information can be challenging and laborious as they require specialized techniques, equipment, and personnel to collect and analyze samples, such as endoscopic evaluation, microbiome analysis, and serological markers. Additionally, the high costs of sample collection, processing, and analysis may limit the use of these study platforms in routine clinical practice. Also, these studies may require long inspection times, which can be a limiting factor for patients and healthcare providers.

Fourier transform–infrared (FT–IR) spectroscopy can provide helpful information about the chemical structures and compositions of biological samples at the molecular level [15]. In the field of gastroenterology, several studies have been conducted on human and animal feces, serum, and colon biopsies using FT–IR and Raman spectroscopy for colitis screening, IBD and cancer diagnosis, and treatment efficacy monitoring [16,17,18,19]. However, to our knowledge, no published study has investigated prediction models for the efficacy of anti-TNF treatment in patients with IBD using fecal samples and FT–IR spectroscopy.

In metabolomics studies, ML algorithms have been widely used to solve data classification and regression problems [20]. For linear data, partial least squares (PLS)–regression or PLS–discriminant analysis (DA) were considered the gold standards for binary classification with their easy interpretation and dimension reduction [21,22]. On the other hand, for nonlinear data, random forest (RF), kernel support vector machines (SVMs), and artificial neural networks, have been suggested for use in clinical metabolomics [23]. In ML, predictors are variables or features used to predict outcomes of interest. Predictors are used to learn the relationships between inputs (features) and outputs (groups) so that the model can make predictions about new or unseen data [24].

Furthermore, the spectral features of fecal samples for predicting anti-TNF treatment efficacy were investigated. The main aim of our study was to explore the application of FT–IR spectroscopy in developing a practical method for predicting treatment outcomes of anti-TNF drugs in a rapid and noninvasive manner.

## 2. Materials and Methods

### 2.1. Participant Recruitment and Study Design

**Figure 1:**
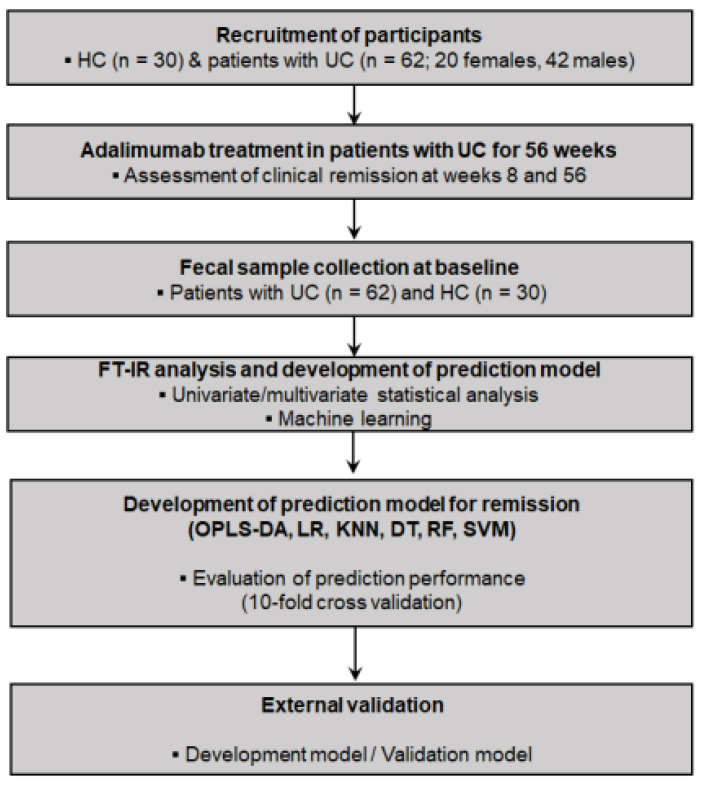
Study design for investigating prediction models (using FT–IR spectroscopy coupled with machine learning algorithms) for clinical remission associated with adalimumab treatment in patients with ulcerative colitis. DT, decision tree; FT–IR, Fourier transform–infrared; HC, healthy controls; KNN, K-nearest neighbors; LR, logistic regression; OPLS–DA, orthogonal partial least squares–discriminant analysis; RF, random forest; SVM, support vector machine; UC, ulcerative colitis.

Adult patients (>18 years of age) were eligible for enrollment in this study. Fecal samples from 62 UC patients and 30 healthy controls (HCs) collected for previous clinical research were used in this study [25]. All UC patients were of moderate to severe active disease status, defined as a Mayo score [26] from 6 to 12, with an endoscopic subscore of at least 2 despite conventional therapy with a regimen based on 5-aminosalicylic acid, corticosteroids, and azathioprine/6-mercaptopurine. Patients with the following clinical features were excluded: malignancy; severe infection, such as active tuberculosis, invasive fungal infection, or opportunistic infection; enrollment in other clinical trials; and pregnancy or breastfeeding. Patients were excluded if they chose to withdraw from the study or were judged to be ineligible by the investigators. The HC group included people without abnormalities among those who visited the hospital for the health screening and provided stool samples. Adalimumab treatment was administered not only as maintenance therapy but also as induction therapy at 160 mg at week 0 and 80 mg at week 2, followed by administration of 40 mg every 2 weeks as maintenance therapy from week 4. In Table 1, 27.4% of patients had already used steroids before starting adalimumab administration. Therefore, the doses of all patients on corticosteroids were tapered to discontinuation within 2 months. Escalating the dosage of adalimumab to 40 mg every week was permitted if patients showed inadequate responses with conventional adalimumab therapy (40 mg every 2 weeks), and discontinuation of adalimumab was also permitted, based on a physician’s judgment, for patients with inadequate responses despite dose escalations [25]. Previous use of anti-TNF-α agents other than adalimumab was permitted if the use had been discontinued due to PNR, LOR, or intolerance. This study was approved by each center’s institutional review board, and written informed consent was obtained from all participants (Chung-Ang University Hospital Institutional Review Board No. C2015020 (approval on 13 March 2015)). The overall workflow of the study is shown in Figure 1.

### 2.2. Patient Assessments

Disease severity was evaluated using the Mayo scoring system. Short-term (at week 8) and long-term (at week 56) clinical remission were evaluated using Mayo scores and a partial Mayo scores according to the following criteria: Mayo score ≤2 points without a subscore >1 point, and partial Mayo score of 0 or 1 point. Patient demographic and anthropometric data (sex, age, and body mass index (BMI)) and baseline clinical characteristics, which included disease-related (disease severity, disease location, and endoscopic findings), laboratory (fecal calprotectin (FC), C-reactive protein (CRP), and serum albumin), and drug history information, were also evaluated before initiating treatment. The full and partial Mayo scores are tools used to evaluate the severity of symptoms in patients with IBD. At 8 weeks, since all patients underwent endoscopy, full Mayo scores were used, and at 56 weeks, more patients did not undergo endoscopy, so partial Mayo scores were used. The full Mayo score is a comprehensive evaluation including endoscopic findings; the partial Mayo score focuses on specific symptoms and does not include endoscopic findings.

### 2.3. Fecal Sample Collection and FT–IR Spectroscopy Analysis

The collected baseline fecal samples from the UC (*n* = 62) and HC (*n* = 30) groups were frozen and stored at −20 °C immediately after collection, and then frozen samples in aluminum foil bags were transferred to the laboratory within 10 min. After thawing at a low temperature (4 °C) to prevent metabolic changes, the samples were diluted 10-fold with phosphate-buffered saline (pH 7.4) and stored at −80 °C for further analysis. Frozen samples were lyophilized for 48 h to remove the strong and broad absorption bands derived from water in fecal samples and to improve the IR intensities of all other components [27]. Dried samples were stored at −80 °C until FT–IR spectroscopy analysis.

Spectroscopy analysis was performed on an FT–IR Nicolet spectrometer (Thermo Scientific, Waltham, MA, USA) equipped with a diamond crystal cell attenuated total reflection (ATR) accessory. Dried samples were loaded onto the ATR crystal and measured under the following analytical conditions: resolution of 4 cm^−1^, 32 scans, and mid-infrared region of 4000–400 cm^−1^. Nine replicates of the instrumental analysis were performed for each sample. To prevent unwanted contamination between samples, the ATR crystal was cleaned with ethanol, and a new background spectrum was measured after measuring each sample. Quality control (QC) samples, which were randomly selected from the HC group, were evaluated after the analysis of every 10 samples to confirm the instrumental stability and analytical reliability of the study.

### 2.4. Spectral Data Processing

The acquired FT–IR spectra were preprocessed using OMNIC9 software (version 9.3.30, Thermo Fisher Scientific, Waltham, MA, USA) to improve spectral interpretation before statistical and ML analyses. Nine replicate spectra from each sample were averaged into three spectra (using the statistical spectra function) for further analysis. These spectra were subjected to baseline correction, which is the attenuated total reflection (ATR) correction algorithm in OMNIC^TM^ 6.2 software for Thermo Scientific Nicolet^TM^ FT-IR spectrometers, to compensate for the effects of variation in the penetration depth of the infrared beam and shift in the infrared absorption band. Derivative spectra were also obtained by applying first and second derivatives (Savitzky–Golay, 7 points, and 3 polynomial order).

The FT–IR spectra had 7201 wavelengths, where each wavelength was considered a feature and all features (predictors) were used for model establishment without feature selection. For statistical and ML analyses, preprocessed FT–IR spectra were converted to comma-separated value files with absorbance values. Four normalization methods (area, min-max, amide, and vector) were applied by manual calculation. In area normalization, the absorbance at each wavenumber was divided by the sum of the total absorbance (for all wavenumbers) of the spectrum in each sample. For min-max normalization, minimum absorbance was subtracted from each absorbance and then divided by the difference between the maximum and minimum absorbance. Amide normalization selected the maximum absorbance in the amide I region (1650–1600 cm^−1^). Thereafter, minimum absorbance (in the entire region) was subtracted from each absorbance and then divided by the difference between the maximum absorbance (amide I region) and minimum absorbance (entire region). For vector normalization, first- and second-derivative spectra were used, and each derivative value was divided by the Euclidean norm [28].

### 2.5. Development of Prediction Models by ML Algorithms

The orthogonal PLS–DA (OPLS–DA) model was established using SIMCA software (version 15, Umetrics, Umeå, Sweden). The optimal components were selected using the autofit function in SIMCA. Good fitness (R^2^Y) and predictability (Q^2^Y) of the model were evaluated (R^2^Y and Q^2^Y values of 1 indicate the perfect model). A permutation test and 10-fold cross-validation (CV) were performed to prevent overfitting of the model. In permutation testing, intercept values of R^2^Y and Q^2^Y below 0.4 and 0.05, respectively, indicate a valid model. A cross-validated analysis of variance test was performed to evaluate the significance of the Q^2^Y (*p* < 0.05).

The OPLS–DA model performance was evaluated in terms of various parameters (accuracy, precision, recall, F1_score, and receiver operating characteristic (ROC]-area under the curve (AUC)) by manual calculation using prediction value (ypred) in SIMCA. Prediction models implying other ML algorithms (logistic regression (LR), K-nearest neighbors (KNN), decision tree (DT), RF, and SVM) were developed using SciKit-Learn software (version 0.24.0) package (Scikit-Learn, http://scikit-learn.org/ (accessed on 24 May 2021); Python Software Foundation, https://www.python.org/ (accessed on 15 September 2021)). The optimal parameters of each method were selected by GridSearchCV in the SciKit-Learn library [29].

The prediction models with various ML algorithms were compared in terms of performance based on accuracy, precision, recall, F1_score, and ROC–AUC values after 10-fold CV. In a 10-fold CV, the whole data set was divided into 10 folds, and the first fold was used for the testing set (10% testing data); the remaining folds were used for the training set (90% training data) [30]. This procedure was repeated 10 times, and the performance results were averaged over the overall results. Accuracy refers to the ratio between the correctly predicted cases and all the cases in the dataset (true positive (TP) and true negative (TN)/true positive (TP) and false negative (FN) and true negative (TN) and false positive (FP)) [31]. Precision and recall (equal to sensitivity) are the proportion of correctly predicted positive cases (TP) to the total predicted positive cases (TP and FP) and to the total true-positive cases (TP and FN), respectively [30]. F1_score is the harmonic mean of precision and recall (2 × (precision × recall)/(precision + recall]) [31]. ROC–AUC analysis is commonly used to evaluate the prediction performance of the ROC curve at various thresholds [32]. 

External validation was performed using various ML algorithms. The entire sample (*n* = 62) was divided into development (*n* = 51) and validation (*n* = 11) samples by collection sites (institutions) because of the limited availability of only a single data set [33]. Detailed information on the samples from the development and validation models is listed in Table S1. The performance of the prediction models (by development samples, *n* = 51) was evaluated by importing external validation samples (*n* = 11). 

Characteristic spectral features for prediction models by OPLS–DA were analyzed using the variable importance of projection cutoff value and univariate statistical analysis, including Student’s *t*-test (SPSS Statistics for Windows, version 25.0, IBM Corp., Armonk, NY, USA) and fold-change analysis (MetaboAnalyst 5.0 (version 5.0), http://www.metaboanalyst.ca/ (accessed on 16 March 2021)).

## 3. Results

### 3.1. Study Population and Baseline Characteristics

The baseline clinical information of the UC group is listed in Table 1. Of the 62 patients, 42 were men, and 20 were women. The mean age was 45.6 years, and the mean BMI was 23.2 kg/m^2^. The mean Mayo and partial Mayo scores were 8.5 and 6.0, respectively. For laboratory tests, the mean FC, CRP, and serum albumin were 668.7 mg/kg, 5.3 mg/dL, and 3.8 g/dL, respectively. Clinical remission rates were 24.2% (based on full Mayo score) and 41.9% (based on partial Mayo score) at weeks 8 and 56, respectively, which were somewhat similar to the findings of our previous studies (24.0% and 41.8% at week 8 and 56, respectively) [25]. 

### 3.2. FT–IR Spectral Assignment of Fecal Samples and Discrimination of HC and Patients with UC

The averaged spectra of fecal samples from the HC and UC groups are shown in Figure S1. The band assignment of the representative 14 peaks was performed, which were associated with proteins, nucleic acids, lipids, and carbohydrates. Detailed information on peak assignment and vibrational modes is listed in Table S2.

**Figure 2:**
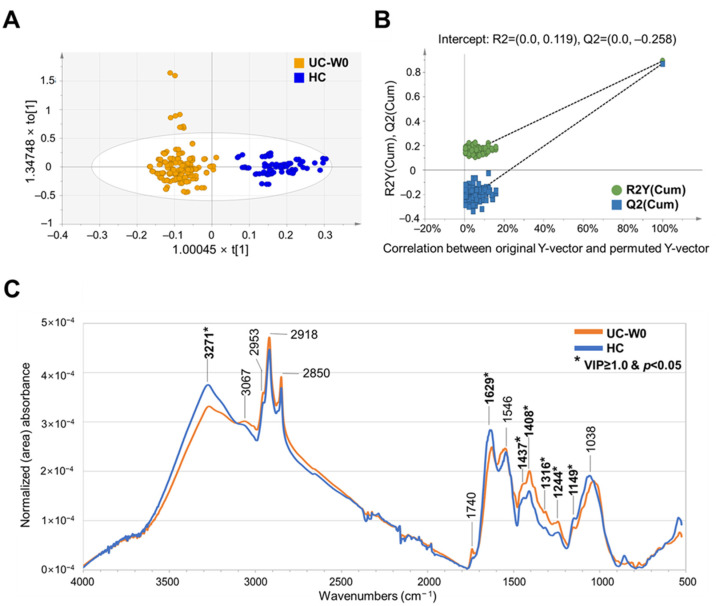
Development of discrimination model and comparison of FT-IR spectral peaks of fecal samples from HC and patients with UC. (**A**) OPLS-DA-derived score plots discriminating fecal samples from HC and patients with UC. t[1] and to[1] in the x- and y-axes represent the predictive (describing between-group variations) and orthogonal (describing within-group variations) component, respectively. (**B**) Permutation test plots of OPLS-DA model with R^2^Y and Q^2^Y intercept values after 100 permutations. (**C**) Comparison of FT-IR spectra of fecal samples from HC and patients with UC applying area normalization method. FT-IR, Fourier transform–infrared; HC, healthy controls; OPLS-DA, orthogonal partial least squares–discriminant analysis; UC, ulcerative colitis. Significant differences between two groups are represented with asterisk mark (*) (*p* < 0.05) in student’s *t* test. Bold characters represent selected wavenumbers satisfying both VIP value over 1.0 and *p*-value (student’s *t* test) below 0.05.

As listed in Table S3, the OPLS–DA model applying area normalization with Pareto scaling yielded the highest R^2^Y (0.890) and Q^2^Y (0.870) values with satisfactory permutation testing (R^2^Y intercept value of 0.119, Q^2^Y intercept value of −0.258). In the OPLS–DA score plots, the two groups were clearly discriminated with valid permutation test plots (Figure 2A,B).

The characteristic peaks discriminating the UC and HC groups were as follows: more intense peaks in UC—1437 cm^−1^, lipid; 1408 cm^−1^, fatty acid and amino acid; 1316 cm^−1^, protein; 1244 cm^−1^, DNA, and more intense peaks in HC—3271 and 1629 cm^−1^, protein; 1149 cm^−1^, carbohydrate (Tables S2 and S4). QC samples were tightly clustered in principal component analysis score plots representing instrumental stability and the reliability of the analysis (Figure 2). Our study identified that more intense peaks associated with proteins (1.37-and 1.38-fold change versus HC), amino acids (1.26-fold change versus HC), lipids (1.20-fold change versus HC), and DNA (1.29-fold change versus HC) were found in fecal samples of UC patients, and less-intense peaks of amino acids (0.99-fold change versus NRM on W56) and more-intense peaks of carbohydrates (1.21-fold change versus NRM on W8, 1.24-fold change versus NRM on W56) were characterized in fecal samples of RM (Table S4).

### 3.3. OPLS–DA-Based Prediction Model for Clinical Remission Associated with Adalimumab Treatment in Patients with UC

**Figure 3:**
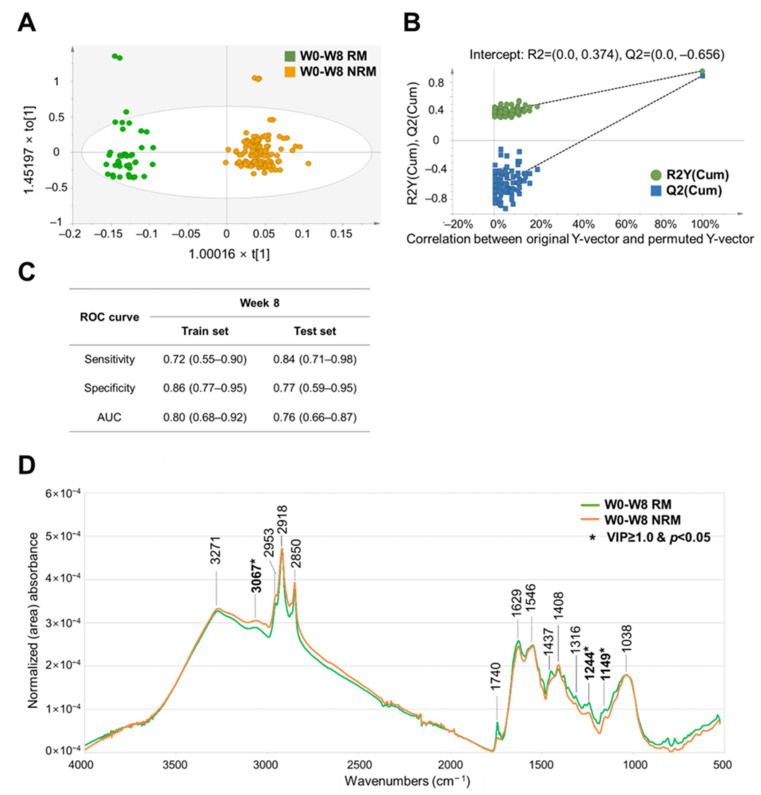
Development of a discrimination model and comparison of FT–IR spectral peaks of baseline fecal samples from RM and NRM at 8 weeks with adalimumab treatment. (**A**) OPLS–DA-derived score plots discriminating baseline fecal samples from RM and NRM at 8 weeks with adalimumab treatment. t[1] and to[1] on the x- and y-axes represent the predictive (describing between-group variations) and orthogonal (describing within-group variations) components, respectively. (**B**) Permutation test plots of the OPLS–DA model with R^2^Y and Q^2^Y intercept values after 100 permutations. (**C**) Sensitivity, specificity, and AUC values in the ROC curve analysis of all spectral data discriminating baseline fecal samples from RM and NRM at 8 weeks with adalimumab treatment. The 95% confidence interval shown in parentheses. (**D**) Comparison of the FT–IR spectra of baseline fecal samples from RM and NRM, applying the area normalization method. AUC, area under the curve; FPR, false-positive rate; FT–IR, Fourier-transform infrared; NRM, patients not in remission; OPLS–DA, orthogonal partial least squares–discriminant analysis; RM, patients in remission; ROC, receiver operating characteristic; TPR, true-positive rate. Significant differences between two groups are represented with asterisk mark (*) (*p* < 0.05) in student’s *t* test. Bold characters represent selected wavenumbers satisfying both VIP value over 1.0 and *p*-value (student’s *t* test) below 0.05.

For short-term remission (8 weeks), the best OPLS–DA model was developed by applying area normalization and Pareto scaling, which had the highest R^2^Y and Q^2^Y values of 0.954 and 0.888, respectively (Table S3). Score plots showed a clear separation between the RM and NRM (Figure 3A). The permutation test was also satisfied with R^2^Y and Q^2^Y intercept values of 0.374 and −0.656, respectively (Figure 3B and Table S3). In the ROC curve analysis, the sensitivity, specificity, and AUC values were 0.84 (95% confidence interval (CI), 0.71–0.98), 0.77 (CI, 0.59–0.95), and 0.76 (CI, 0.66–0.87), respectively, in the test set after 10-fold CV representing acceptable performance (Figure 3C).

**Figure 4:**
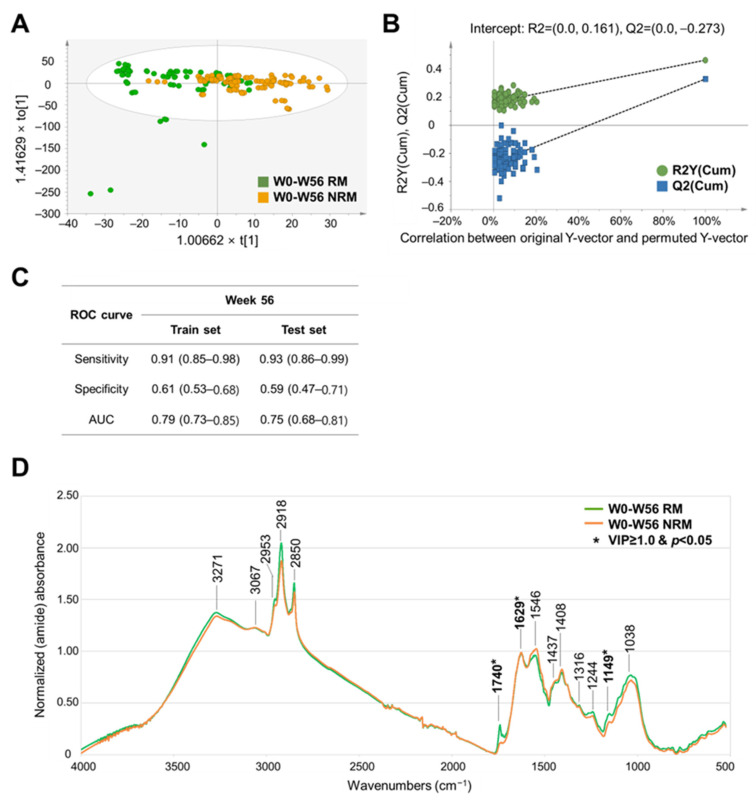
Development of a discrimination model and comparison of FT–IR spectral peaks of baseline fecal samples from RM and NRM at 56 weeks with adalimumab treatment. (**A**) OPLS–DA-derived score plots discriminating baseline fecal samples from RM and NRM at 56 weeks with adalimumab treatment. t[1] and to[1] on the x- and y-axes represent the predictive (describing between-group variations) and orthogonal (describing within-group variations) components, respectively. (**B**) Permutation test plots of the OPLS–DA model with R^2^Y- and Q^2^Y-intercept values after 100 permutations. (**C**) Sensitivity, specificity, and AUC values in the ROC curve analysis of all spectral data discriminating baseline fecal samples from RM and NRM at 56 weeks with adalimumab treatment. The 95% confidence interval shown in parentheses. (**D**) Comparison of the FT–IR spectra of baseline fecal samples from RM and NRM at 56 weeks with adalimumab treatment by applying the amide normalization method. AUC, area under the curve; FPR, false-positive rate; FT–IR, Fourier transform–infrared; NRM, patients not in remission; OPLS–DA, orthogonal partial least squares–discriminant analysis; RM, patients in remission; ROC, receiver operating characteristic; TPR, true-positive rate. Significant differences between two groups are represented with asterisk mark (*) (*p* < 0.05) in student’s *t* test. Bold characters represent selected wavenumbers satisfying both VIP value over 1.0 and *p*-value (student’s *t* test) below 0.05.

For long-term remission (56 weeks), the best OPLS–DA model was selected by applying amide normalization and unit variance scaling with R^2^Y and Q^2^Y values of 0.461 and 0.327, respectively (Table S3). Score plots showed a clear separation between the RM and NRM (Figure 4A). The permutation test was also satisfied with R^2^Y and Q^2^Y intercept values of 0.161 and −0.273, respectively (Figure 4B and Table S3). The ROC curve analysis showed acceptable performance of the model, with sensitivity, specificity, and AUC values of 0.93 (CI, 0.86–0.99), 0.59 (CI, 0.47–0.71), and 0.75 (CI, 0.68–0.81), respectively, in the test set after 10-fold CV (Figure 4C).

Variable influence on projection (VIP) was used to investigate the peaks that contribute most to the OPLS-DA prediction model (Table S4). For short-term remission (8 weeks), peaks associated with proteins (VIP value: 1.46), DNA (VIP value: 1.68), and carbohydrates (VIP value: 1.60) were characterized as contributing to the discrimination between RM and NRM. For long-term remission (56 weeks), peaks associated with triacylglycerol (VIP value: 1.46), protein (VIP value: 1.52, 1.60), and carbohydrate (VIP value: 2.72, 2.70) were characterized as contributing to the discrimination between RM and NRM. These characteristic spectral peaks (with VIP value cutoff of 1.0 or higher and a *p*-value of less than 0.05) from fecal samples by remission period have the potential to be used as biomarkers for predicting short- and long-term remission.

### 3.4. Comparison of the Prediction Performance of Various ML Algorithms

Prediction models for short-term (8 weeks) and long-term (56 weeks) remission were established using the entire sample (*n* = 62) by employing various ML algorithms, and the prediction performance of the models after 10-fold CV was compared (Table 2 and Table S5). Notably, the ML algorithms used in the best-performing short-term (8 weeks) and long-term (56 weeks) remission prediction models are listed in Table 2. For short-term remission, the model with the best predictive performance on the test set was developed by applying LR and radial basis function (rbf) SVMs with an accuracy of 0.99 (95% confidence interval (CI), 0.98–1.01) (Table 2). Linear SVM was also a good algorithm to be applied for predicting short-term remission (accuracy of 0.97 (CI 0.94–1.01) in the test set) (Table S5). For long-term remission, the best prediction model was developed by rbf-SVM, revealing 0.99 (CI 0.98–1.01) in the test set (Table 2). LR, KNN, and linear SVM also showed excellent performance for predicting long-term remission (accuracy of 0.96 (CI 0.90–1.02], 0.96 (CI 0.92–1.00), and 0.96 (CI 0.91–1.01), respectively, in the test set) (Table S5). Whereas DT, RF, and OPLS–DA showed relatively poor performance (Table S5).

Prediction models applying LR (for short-term remission) and DT (for long-term remission) were selected as the optimal models for external validation (Table 3 and Table S6). These models showed excellent and good performance in the internal (using development samples, *n* = 51) and external validation (using validation samples, *n* = 11) of each model (Table 3). As listed in Table 3, the accuracy, precision, recall, F1_score, and ROC–AUC values for short-term remission models (by LR) were all above 0.7 when importing validation samples (*n* = 11), which represents good performance. For long-term remission, the values of accuracy, prediction, recall, and F1_score were above 0.8 in the prediction model by DT, and the AUC value was 0.69 in external validation samples (*n* = 11) (Table 3).

## 4. Discussion

Determining whether to use anti-TNF agents is a critical issue in the management of UC. PNR or secondary LOR can make the patient’s treatment more complex and undermine their quality of life [34]. For this reason, many attempts have been made to predict the efficacy of these drugs using various parameters before treatment. However, unsatisfactory results and lost time have hindered their clinical applications. Missing the optimal timing for the administration of anti-TNF treatment can be associated with worsened disease status. Thus, the ideal biomarker probably meets two conditions: reliability and a suitable processing time. This study established a prediction model for the treatment efficacy of anti-TNF drugs in a noninvasive, easy, simple, and rapid way with the application of FT–IR spectroscopy for use in clinical practice.

The prediction models for short-term (by LR or rbf-SVM) and long-term (by rbf-SVM and DT) remission showed good performance in our study. In IBD research, LR, SVM, and DT have been widely used to predict disease progression, course, risk factors, and treatment outcomes [35,36,37,38,39,40]. LR is a statistical model in which the probability of the outcome variable (dependent variables, Y) is predicted by the sigmoid function as a linear combination of potential predictor variables (independent variables, X) [40]. It is helpful in modeling medical problems because of the well-established methodology and intuitive clinical interpretation of coefficients [41]. SVM attempts to find the class boundary between two different classes through an optimal hyperplane that divides the data into two classes while maximizing the marginal distance of the two classes and minimizing classification errors [32,42]. For a nonlinear data set, a kernel trick can be used in SVM, which maps the input data into a higher-dimensional space and makes the separation easier [21]. DT provides high classification accuracy through a reliable and effective decision-making technique with simple representations of collected knowledge [43]. It has been widely used in various decision-making areas in the medical field [43]. It is known as an intuitive binary logic-based predictive classification algorithm for multivariate analysis [21]. Meanwhile, the OPLS–DA algorithm performed relatively poorly in our study; thus, it is expected that nonlinear ML algorithms could be used as more suitable strategies for developing prediction models.

When developing a prognostic and diagnostic prediction model, internal validation is necessary to evaluate any optimism in the model, and external validation (using other sample sets rather than for model development) is also recommended [33]. Prediction models for short-term (by LR or rbf-SVM) and long-term (by rbf-SVM and DT) remission in our study performed well for both internal validation (using the entire sample) and external validation. For long-term remission, different algorithms, such as rbf-SVM and DT, were, respectively, applied in our study to develop prediction models for internal and external validation, probably due to the small sample size. Further studies are needed to investigate and optimize prediction models better suited for long-term remission by employing more samples collected from independent development and validation cohorts to improve the reliability and robustness of the models.

As listed in Table S4, UC patients had more intense peaks with amino acids (1.26-fold change versus HC) in their fecal samples, which is consistent with several studies reporting that fecal amino acids are associated with CD severity. There was also an intense peak of carbohydrates (1.60-fold change versus NRM on W8, 1.24-fold change versus NRM on W56) in RM patients, which is consistent with the finding of enhanced carbohydrate metabolism in the gut microbiome of a mouse model of IBD. Similar findings have been reported elsewhere, indicating that fecal amino acids are positively correlated with increased CD severity [44], and carbohydrate metabolism was enriched in the gut microbiome in an IBD mouse model with treatment-induced remission [45].

In an FT–IR analysis with an ATR technique, some challenges have been reported: maintaining an intimate optical contact area between the sample and the crystal surface [46], as well as problems concerning homogeneity and granulometry [47] derived from nonuniform particles of powdered samples. In our study, these challenges were overcome by using an FT–IR-ATR instrument equipped with a pressure clamp, nine replicates of instrumental analysis, analysis of QC samples, and data preprocessing steps (ATR correction and normalization-scaling). Among these, data preprocessing is crucial to compensate for experimental bias, as IR data are easily affected by subtle changes in experimental conditions or spectroscopy settings and can be distorted by sample-unrelated noise [48]. Normalization is helpful for reducing the peak intensity variation originating from various sample thicknesses and pathlength variations in FT–IR analysis [49,50]. Scaling is used to reduce the intensity variation between spectral peaks within the sample, which makes all spectral peaks equally important to the spectrum [51]. These strategies could be suggested as standardized operation procedures of FT–IR analysis, including sampling, experimental analysis, and data preprocessing, which can be applied to real clinical settings with high reproducibility.

However, this study had some limitations. The small sample size limits the reliability and robustness of the models. Further studies with larger samples are needed to validate the prediction models for long-term remission. In conclusion, to our knowledge, this was the first study to investigate human fecal samples using FT–IR spectroscopy combined with ML algorithms to develop a prediction model for clinical remission associated with adalimumab treatment in patients with UC. With noninvasive, simple, inexpensive, fast, and reliable analysis, FT–IR spectroscopy coupled with ML algorithms may be used in real clinical settings to help clinicians select patients who are likely to respond to treatment.

## Figures and Tables

**Table 1:** Demographics and baseline clinical characteristics of patients with UC.

Characteristic	Patients with UC (*n* = 62)
**Female/Male, *n***	20/42
**Age, mean ± SD, years**	45.6 ± 14.9
**Body mass index, mean ± SD, kg/m^2^**	23.2 ± 3.9
**Mayo score, mean ± SD**	8.5 ± 1.3
**Partial Mayo score, mean ± SD**	6.0 ± 1.2
**Endoscopic finding, *n* (%)**	
Moderate	33 (53.0)
Severe	29 (47.0)
**Disease location, *n* (%)**	
Proctitis	13 (21.0)
Left-sided colitis	29 (47.0)
Extensive colitis	20 (32.0)
**Fecal calprotectin, mg/kg**	
Mean ± SD	668.7 ± 509.5
Median	543.1
**C-reactive protein, mg/dL**	
Mean ± SD	5.3 ± 14.2
Median	0.9
IQR	3 (0.19–3.19)
**Albumin, g/dL**	
Mean ± SD	3.8 ± 0.6
Median	4.0
**Concomitant medication (overlapped), *n* (%)**	
5-Aminosalicylate	51 (82.3)
Methotrexate	2 (3.2)
Azathioprine/6-Mercaptopurine	30 (48.4)
Systemic corticosteroid	17 (27.4)
**Prior anti-tumor necrosis factor therapy, *n* (%)**	
1 medication	12 (19.4)
≥2 medications	0 (0)

IQR, interquartile range; SD, standard deviation; UC, ulcerative colitis. All values are mean ± standard deviation unless otherwise noted.

**Table 2:** Best ML algorithm with 10-fold cross-validation performance using baseline fecal samples for remission prediction models at 8 and 56 weeks of adalimumab treatment in patients with UC.

Week 8	Parameters	Accuracy		Precision		Recall		F1_Score		ROC–AUC	
Methods		Train	Test	Train	Test	Train	Test	Train	Test	Train	Test
LR	C = 1	1.00	0.99 (0.98–1.01)	1.00	1.00	1.00	0.98 (0.93–1.03)	1.00	0.99 (0.96–1.01)	1.00	0.99 (0.97–1.01)
rbf SVM	kernel = ‘rbf’, gamma = 0.0001, C = 100	1.00	0.99 (0.98–1.01)	1.00	1.00	1.00	0.98 (0.92–1.03)	1.00	0.99 (0.95–1.02)	1.00	0.99 (0.96–1.02)
Week 56	Parameters	Accuracy		Precision		Recall		F1_score		ROC–AUC	
Methods		Train	Test	Train	Test	Train	Test	Train	Test	Train	Test
rbf SVM	kernel = rbf, gamma = 0.0001, C = 1000	1.00	0.99 (0.98–1.01)	1.00	0.99 (0.95–1.02)	1.00	1.00	1.00	0.99 (0.97–1.01)	1.00	0.99 (0.99–1.00)

rbf, radial basis function; SVM, support vector machine; UC, ulcerative colitis. LR and SVM were performed by SciKit-Learn software (version 0.24.0) and parameters were selected by the function of “GridSearchCV” in SciKit-Learn software (version 0.24.0). The 95% confidence intervals are presented within parentheses.

**Table 3:** Performance characteristics (for external validation) of machine learning models to predict short-term (week 8) and long-term (week 56) remission associated with adalimumab treatment in patients with ulcerative colitis.

Evaluators	Week 8 (LR)			Week 56 (DT)		
	Development Model (*n* = 51)		Validation Model (*n* = 11)	Development Model (*n* = 51)		Validation Model (*n* = 11)
	Train (95% CI)	Test (95% CI)		Train (95% CI)	Test (95% CI)	
Accuracy	1.00	0.99 (0.98–1.01)	0.73	0.99 (0.99–1.00)	0.90 (0.84–0.96)	0.82
Precision	1.00	1.00	0.72	1.00	0.88 (0.77–0.98)	0.82
Recall	1.00	0.95 (0.84–1.06)	0.73	0.99 (0.99–1.00)	0.93 (0.87–0.98)	0.82
F1_score	1.00	0.97 (0.89–1.04)	0.72	0.99 (0.99–1.00)	0.89 (0.82–0.96)	0.82
ROC–AUC	1.00	0.98 (0.92–1.03)	0.75	0.99 (0.99–1.00)	0.91 (0.85–0.96)	0.69

95% confidence intervals are indicated within parentheses, with the parameter values in the development model. CI, confidence interval; LR, logistic regression; ROC–AUC, receiver operating characteristic–area under the curve; DT, decision tree.

## Data Availability

The data that support the findings of this study are available from the corresponding author upon reasonable request. Data are not publicly available due to privacy.

## Supplementary Materials

The following supporting information can be downloaded at https://www.mdpi.com/article/10.3390/metabo14010002/s1, Figure S1. Averaged Fourier transform–infrared (FT–IR) spectrum of baseline fecal samples from healthy controls and patients with ulcerative colitis. Table S1. Institutional and collection data information of samples from the development and validation models. A, The Catholic University of Korea St. Vincent’s Hospital; B, Chung-Ang University Hospital; C, Seoul National University Hospital; D, SNU Boramae Medical Center; E, Ewha Womans University Mokdong Hospital; F, Chosun University Hospital; G, Daejeon St. Mary’s Hospital; H, Severance Hospital; I, Keimyung University Dongsan Medical Center; J, Korea University Anam Hospital; K, Inje University Seoul Paik Hospital; L, Inje University Haeundae Paik Hospital; M, Chonnam National University Hospital; N, Kangbuk Samsung Hospital; O, Inha University Hospital; P, Kyungpook National University Hospital; Q, KyungHee University Medical Center. Table S2. Assignment of FT–IR spectra of fecal samples from patients with ulcerative colitis and healthy controls. ^(a)^ Detailed information on each reference used for spectral assignment is listed in the reference section below. Table S3. Parameters of OPLS–DA models applying various normalization and scaling methods for discriminating baseline fecal samples in three comparison cases. CV-ANOVA, cross validated-analysis of variance; HC, healthy controls; OPLS-DA, orthogonal partial least squares-discriminant analysis; UC, ulcerative colitis; UV, unit variance; RM, patients in remission; NRM, patients not in remission; W8, baseline fecal samples from patients after 8 weeks of adalimumab treatment; W56, baseline fecal samples from patients after 56 weeks of adalimumab treatment. Bold characters represent the best parameters of the selected model. Table S4. Comparison of absorbance intensity of major assigned spectral peaks from fecal samples in three types of discrimination models. HC, healthy controls; NRM, patient not in remission; RM, patient in remission; UC, ulcerative colitis; VIP, variable importance of projection; W8, baseline fecal samples from patients after 8 weeks of adalimumab treatment; W56, baseline fecal samples from patients after 56 weeks of adalimumab treatment. The Direction of comparison is UC/HC and RM/NRM with a fold change threshold of 1.0. Significant differences between the two groups are indicated with asterisks (*) (*p* < 0.05, Student’s *t*-test. Bold characters represent selected wavenumbers satisfying both VIP values over 1.0 and *p*-values (Student’s *t*-test) below 0.05. Table S5. Comparison of 10-fold cross-validation performance, by various machine learning algorithms using baseline fecal samples, of the remission prediction model at 8 and 56 weeks of adalimumab treatment in patients with UC. DT, decision tree; KNN, K-nearest neighbors; LR, logistic regression; OPLS–DA, orthogonal partial least squares–discriminant analysis; RF, random forest; ROC–AUC, receiver operating characteristic–area under the curve; SVM, support vector machine; UC, ulcerative colitis. LR, KNN, DT, RF, and SVM were performed by SciKit-Learn software (version 0.24.0) and parameters were selected by the function of “GridSearchCV” in SciKit-Learn software (version 0.24.0). OPLS–DA was performed using SIMCA software (version 15.0.2), and the parameters were selected using the “autofit” function in SIMCA. The 95% confidence intervals are presented within parentheses. Table S6. Comparison of the predictive performances of various ML algorithms (with internal and external validation) of the remission prediction model at 8 and 56 weeks of adalimumab treatment in patients with ulcerative colitis. The 95% confidence intervals are indicated within parentheses, with the parameter values in the development model. DT, decision tree; KNN, K-nearest neighbors; LR, logistic regression; rbf, radial basis function; RF, random forest; ROC–AUC, receiver operating characteristic-area under the curve; SVM, support vector machine; UC, ulcerative colitis. LR, KNN, DT, RF, and SVM were performed by SciKit-Learn software (version 0.24.0) and parameters were selected by the function of “GridSearchCV” in SciKit-Learn software (version 0.24.0).
