# Supplementary material for: Prediction of Clinical Remission with Adalimumab Therapy in Patients with Ulcerative Colitis by Fourier Transform–Infrared Spectroscopy Coupled with Machine Learning Algorithms
Source: Metabolites. 2023 Dec 19;14(1):2. doi: 10.3390/metabo14010002 (PMC10818421; doi:10.3390/metabo14010002)
Supplement: Supplementary file 1 [file metabolites-14-00002-s001.zip › Table S1.pdf]

Table S1. Institute and collection data information of samples from development and validation model.

Development model						Validation model	
Institutes	Collection date	Institutes	Collection date	Institutes	Collection date	Institutes	Collection date
A (6)	2015-06-05	D (4)	2015-08-04	I (1)	2015-06-23	Q (11)	2015-09-24
	2015-10-28		2016-07-21	J (1)	2017-01-03		2015-12-15
	2015-11-18		2016-12-26	K (3)	2015-09-10		2015-12-22
	2016-06-14		2017-01-05		2016-02-12		2015-12-17
	2016-06-16	E (3)	2015-10-12		2016-11-22		2015-12-29
	2017-01-03		2016-01-19	L (6)	2015-12-08		2016-06-10
B (8)	2015-06-29		2016-07-05		2016-01-14		2016-06-21
	2015-07-27	F (2)	2016-05-04		2016-01-22		2016-06-24
	2015-11-17		2016-04-27		2016-06-30		2016-06-18
	2016-03-08	G (3)	2016-01-19		2016-11-23		2016-08-23
	2016-04-07		2016-05-03		2017-02-16		2017-02-21
	2016-10-31		2016-06-22	M (1)	2016-11-11		
	2016-12-03	H (5)	2015-07-03	N (1)	2016-06-16		
	2017-06-03		2016-03-28	O (2)	2016-07-26		
C (3)	2015-09-09		2016-04-01		2016-08-23		
	2016-10-11		2016-05-31	P (2)	2016-05-27		
	2017-02-22		2017-02-08		2017-03-07		

A, The Catholic University of Korea St. Vincent’s Hospital; B, Chung-Ang University Hospital; C, Seoul National University Hospital; D, SNU Boramae Medical Center; E, Ewha Womans University Mokdong Hospital; F, Chosun University Hospital; G, Daejeon St. Mary’s Hospital; H, Severance Hospital; I, Keimyung University Dongsan Medical Center; J, Korea University Anam Hospital; K, Inje University Seoul Paik Hospital; L, Inje University Haeundae Paik Hospital; M, Chonnam National University Hospital; N, Kangbuk Samsung Hospital; O, Inha University Hospital; P, Kyungpook National University Hospital; Q, KyungHee University Medical Center.
