# Supplementary material for: Prediction of Clinical Remission with Adalimumab Therapy in Patients with Ulcerative Colitis by Fourier Transform–Infrared Spectroscopy Coupled with Machine Learning Algorithms
Source: Metabolites. 2023 Dec 19;14(1):2. doi: 10.3390/metabo14010002 (PMC10818421; doi:10.3390/metabo14010002)
Supplement: Supplementary file 1 [file metabolites-14-00002-s001.zip › Table S2.pdf]

Table S2. Assignment of FT-IR spectra of fecal samples from patients with ulcerative colitis and healthy controls.

No.	Wavenumber (cm^{-1})	Assignments	Vibrational mode	Reference[a)]
1	3271	Amide A, proteins	N–H stretching	(1)
2	3067	Amide B, proteins	N–H stretching	(1)
3	2953	Lipids, proteins	CH_3 asymmetric stretch	(1)
4	2918	Saturated lipids, side chain of proteins	CH_2 asymmetric stretch	(1)
5	2850	Saturated lipids and side chain of proteins	CH_2 symmetric stretch	(1)
6	1740	Triglycerides, glycerol esters	Carbonyl C=O stretch	(2)
7	1629	Amide I, proteins	α-helical, β-pleated sheet, β-turns, random coils and side-chain structures	(3)
8	1546	Amide II, proteins	N–H bend, C–N stretch	(2)
9	1437	Lipid	CH_2 deformation	(4)
10	1408	Fatty acids, amino acids	COO^- symmetric stretch	(1)
11	1316	Amide III, proteins	C–H/N–H deformation vibrational modes methyl group	(5)
12	1244	DNA	Asymmetric PO_2^- stretch	(1)
13	1149	Carbohydrates	C–O stretching mode	(6)
14	1038	Carbohydrate, β-anomer	C–O stretching	(5)

[a)] Detailed information on each reference used for spectral assignment is listed in the reference section below.
