# Supplementary material for: Prediction of Clinical Remission with Adalimumab Therapy in Patients with Ulcerative Colitis by Fourier Transform–Infrared Spectroscopy Coupled with Machine Learning Algorithms
Source: Metabolites. 2023 Dec 19;14(1):2. doi: 10.3390/metabo14010002 (PMC10818421; doi:10.3390/metabo14010002)
Supplement: Supplementary file 1 [file metabolites-14-00002-s001.zip › Table S3.pdf]

Table S3. Parameters of OPLS–DA models applying various normalization and scaling methods for discriminating baseline fecal samples in three comparison cases.

Models	Parameters	Normalization methods				
		**Area**	**Min-max**	**Amide**	**Vector (1st)**	**Vector (2nd)**
HC vs. UC	Scaling method	**Par**	UV	Par	Par	UV
	Component number (orthogonal + predictive)	**1+10**	1+11	1+8	1+8	1+5
	R^2Y	**0.890**	0.890	0.857	0.986	0.989
	Q^2Y	**0.870**	0.860	0.834	0.969	0.959
	R^2Y intercept	**0.119**	0.15	0.087	0.46	0.681
	Q^2Y intercept	**−0.258**	−0.284	−0.207	−0.606	−0.732
	CV-ANOVA (*p*-value)	**0**	0	0	0	0
RM vs. NRM (W8)	Scaling method	**Par**	Par	UV	Par	UV
	Component number (orthogonal + predictive)	**1+21**	1+6	1+8	1+10	1+6
	R^2Y	**0.954**	0.504	0.553	0.985	0.981
	Q^2Y	**0.888**	0.408	0.457	0.947	0.915
	R^2Y intercept	**0.374**	0.118	0.158	0.606	0.786
	Q^2Y intercept	**−0.656**	−0.211	−0.277	−0.801	−0.659
	CV-ANOVA (*p*-value)	**0**	1.22E-13	1.34E-14	0	0
RM vs. NRM (W56)	Scaling method	UV	UV	**UV**	UV	UV
	Component number (orthogonal + predictive)	1+7	1+8	**1+8**	1+8	1+6
	R^2Y	0.402	0.428	**0.461**	0.983	0.983
	Q^2Y	0.268	0.274	**0.327**	0.929	0.922
	R^2Y intercept	0.156	0.175	**0.161**	0.699	0.783
	Q^2Y intercept	−0.247	−0.271	**−0.273**	−0.712	−0.661
	CV-ANOVA (*p*-value)	3.69E−06	8.73E−06	**6.05E−08**	0	0

CV-ANOVA, cross validated-analysis of variance; HC, healthy controls; OPLS-DA, orthogonal partial least squares-discriminant analysis; UC, ulcerative colitis; UV, unit variance; RM, patients in remission; NRM, patients not in remission. W8, baseline fecal samples from patients after 8 weeks of adalimumab treatment; W56, baseline fecal samples from patients after 56 weeks of adalimumab treatment. Bold characters represent the best parameters of the selected model.
