# Supplementary material for: Prediction of Clinical Remission with Adalimumab Therapy in Patients with Ulcerative Colitis by Fourier Transform–Infrared Spectroscopy Coupled with Machine Learning Algorithms
Source: Metabolites. 2023 Dec 19;14(1):2. doi: 10.3390/metabo14010002 (PMC10818421; doi:10.3390/metabo14010002)
Supplement: Supplementary file 1 [file metabolites-14-00002-s001.zip › Table S4.pdf]

**Table S4. Comparison of absorbance intensity of major assigned spectral peaks from fecal samples in three types of discrimination models.**

| Wavenumber (cm$^{-1}$) | UC vs. HC | | | Wavenumber (cm$^{-1}$) | RM vs. NRM (W8) | | | Wavenumber (cm$^{-1}$) | RM vs. NRM (W56) | | |
|---|---|---|---|---|---|---|---|---|---|---|---|
| | VIP value | Fold change (UC/HC) | *p*-value | | VIP value | Fold change (RM/NRM) | *p*-value | | VIP value | Fold change (RM/NRM) | *p*-value |
| **3271.65** | 1.99 | 0.88(↓) | **0.000*** | **3067.23** | 1.46 | 0.95(↓) | **0.048*** | **1740.92** | 1.46 | 2.50(↑) | **0.044*** |
| **3271.16** | 1.99 | 0.88(↓) | **0.000*** | **3067.71** | 1.46 | 0.95(↓) | **0.048*** | **1740.44** | 1.46 | 2.46(↑) | **0.043*** |
| **1629.56** | 1.74 | 0.88(↓) | **0.000*** | 2953.45 | 1.25 | 0.95(↓) | 0.180 | **1629.56** | 1.52 | 0.99(↓) | **0.014*** |
| **1629.07** | 1.71 | 0.88(↓) | **0.000*** | 2953.93 | 1.24 | 0.95(↓) | 0.188 | **1629.07** | 1.60 | 0.99(↓) | **0.019*** |
| **1437.67** | 1.53 | 1.20(↑) | **0.000*** | 1740.44 | 1.76 | 2.09(↑) | 0.226 | 1546.63 | 1.12 | 0.93(↓) | 0.069 |
| **1437.19** | 1.55 | 1.20(↑) | **0.000*** | 1740.92 | 1.74 | 2.06(↑) | 0.226 | 1546.15 | 1.15 | 0.93(↓) | 0.076 |
| **1408.75** | 2.09 | 1.26(↑) | **0.000*** | 1316.18 | 1.35 | 1.14(↑) | 0.086 | 1244.82 | 1.23 | 1.10(↑) | 0.061 |
| **1408.26** | 2.09 | 1.26(↑) | **0.000*** | 1316.66 | 1.33 | 1.14(↑) | 0.089 | 1244.34 | 1.23 | 1.10(↑) | 0.060 |
| **1316.66** | 1.78 | 1.37(↑) | **0.000*** | **1244.34** | 1.68 | 1.23(↑) | **0.038*** | **1149.37** | 2.72 | 1.24(↑) | **0.000*** |
| **1316.18** | 1.78 | 1.38(↑) | **0.000*** | **1244.83** | 1.68 | 1.22(↑) | **0.038*** | **1149.85** | 2.70 | 1.24(↑) | **0.000*** |
| **1244.83** | 1.25 | 1.29(↑) | **0.000*** | **1149.37** | 1.60 | 1.21(↑) | **0.032*** | | | | |
| **1244.34** | 1.25 | 1.29(↑) | **0.000*** | **1149.85** | 1.60 | 1.21(↑) | **0.033*** | | | | |
| **1149.85** | 1.15 | 0.82(↓) | **0.000*** | | | | | | | | |
| **1149.37** | 1.17 | 0.82(↓) | **0.000*** | | | | | | | | |

HC, healthy controls; NRM, patient not in remission; RM, patient in remission; UC, ulcerative colitis; VIP, variable importance of projection; W8, baseline fecal samples from patients after 8 weeks of adalimumab treatment; W56, baseline fecal samples from patients after 56 weeks of adalimumab treatment.

Direction of comparison is UC/HC and RM/NRM with fold change threshold of 1.0. Significant differences between two groups are represented with asterisk mark (*) ($p<0.05$) in student's *t* test. Bold characters represent selected wavenumbers satisfying both VIP value over 1.0 and *p*-value (student's *t* test) below 0.05.
