# Supplementary material for: Prediction of Clinical Remission with Adalimumab Therapy in Patients with Ulcerative Colitis by Fourier Transform–Infrared Spectroscopy Coupled with Machine Learning Algorithms
Source: Metabolites. 2023 Dec 19;14(1):2. doi: 10.3390/metabo14010002 (PMC10818421; doi:10.3390/metabo14010002)
Supplement: Supplementary file 1 [file metabolites-14-00002-s001.zip › Table S5.pdf]

Table S5. Comparison of 10-fold cross-validation performance, by various machine learning algorithms using baseline fecal samples, of the remission prediction model at 8 and 56 weeks of adalimumab treatment in patients with UC.

Week 8	Parameters	Accuracy		Precision		Recall		F1_score		ROC–AUC	
**Methods**		**Train**	**Test**	**Train**	**Test**	**Train**	**Test**	**Train**	**Test**	**Train**	**Test**
KNN	· K=3	0.99 (0.99–0.99)	0.94 (0.89–0.99)	0.99 (0.99–1.00)	0.92 (0.79–1.05)	0.96 (0.95–0.98)	0.83 (0.70–0.97)	0.98 (0.97–0.99)	0.85 (0.75–0.96)	0.98 (0.97–0.99)	0.90 (0.83–0.97)
DT	· max_depth=6,	0.99 (0.99–1.00)	0.88 (0.82–0.95)	1.00	0.80 (0.62–0.99)	0.99 (0.96–1.01)	0.76 (0.56–0.96)	0.99 (0.98–1.00)	0.72 (0.56–0.89)	0.99 (0.98–1)	0.84 (0.75–0.94)
RF	· n_estimators=200, max_depth=4, min_samples_leaf=8, min_samples_split=20	0.94 (0.93–0.94)	0.84 (0.79–0.89)	1.00	0.84 (0.59–1.09)	0.73 (0.70–0.77)	0.41 (0.17–0.66)	0.85 (0.82–0.87)	0.47 (0.28–0.67)	0.87 (0.85–0.88)	0.70 (0.59–0.81)
linear SVM	· C=0.01	1.00	0.97 (0.94–1.01)	1.00	0.94 (0.80–1.08)	1.00	0.96 (0.89–1.02)	1.00	0.93 (0.83–1.03)	1.00	0.97 (0.93–1.01)
OPLS–DA	· Component number=1+21	0.84 (0.79–0.90)	0.82 (0.76–0.87)	0.84 (0.78–0.90)	0.81 (0.76–0.87)	0.99 (0.99–1.00)	0.99 (0.98–1.01)	0.91 (0.88–0.94)	0.89 (0.86–0.92)	0.80 (0.68–0.92)	0.76 (0.66–0.87)
**Week 56**	**Parameters**	**Accuracy**		**Precision**		**Recall**		**F1_score**		**ROC–AUC**	
**Methods**		**Train**	**Test**	**Train**	**Test**	**Train**	**Test**	**Train**	**Test**	**Train**	**Test**
LR	· C=1000	1.00	0.96 (0.9–1.02)	1.00	0.96 (0.87–1.05)	1.00	0.96 (0.88–1.05)	1.00	0.95 (0.88–1.02)	1.00	0.97 (0.91–1.02)
KNN	· K=3	0.99 (0.99–1.00)	0.96 (0.92–1.00)	0.99 (0.99–1.00)	0.95 (0.87–1.03)	0.98 (0.98–0.99)	0.96 (0.92–1.01)	0.99 (0.98–0.99)	0.95 (0.89–1.01)	0.99 (0.98–0.99)	0.97 (0.93–1.00)
DT	· max_depth=5	0.98 (0.96–1.01)	0.88 (0.83–0.93)	1.00	0.92 (0.86–0.98)	0.96 (0.91–1.02)	0.81 (0.69–0.92)	0.98 (0.95–1.01)	0.85 (0.77–0.92)	0.98 (0.95–1.01)	0.87 (0.82–0.93)
RF	· n_estimators=100, max_depth=4, min_samples_leaf=8, min_samples_split=8	0.97 (0.97–0.98)	0.85 (0.80–0.90)	1.00	1.00	0.94 (0.92–0.96)	0.65 (0.56–0.74)	0.97 (0.96–0.98)	0.78 (0.72–0.85)	0.97 (0.96–0.98)	0.83 (0.78–0.87)
linear SVM	· C=0.1	1.00	0.96 (0.91–1.01)	1.00	0.97 (0.89–1.04)	1.00	0.95 (0.86–1.03)	1.00	0.95 (0.88–1.01)	1.00	0.96 (0.91–1.01)
OPLS–DA	· Component number=1+7	0.76 (0.72–0.80)	0.70 (0.63–0.76)	0.75 (0.72–0.78)	0.71 (0.66–0.76)	0.90 (0.86–0.93)	0.80 (0.71–0.90)	0.82 (0.78–0.85)	0.75 (0.69–0.81)	0.79 (0.73–0.85)	0.75 (0.68–0.81)

DT, decision tree; KNN, K-nearest neighbors; LR, logistic regression; OPLS–DA, orthogonal partial least squares–discriminant analysis; rbf, radial basis function; RF, random forest; ROC–AUC, receiver operating characteristic-area under the curve; SVM, support vector machine; UC, ulcerative colitis. LR, KNN, DT, RF, and SVM were performed by SciKit-Learn software and parameters

were selected by the function of "GridSearchCV" in SciKit-Learn software. OPLS–DA was performed using SIMCA software, and the parameters were selected using the "autofit" function in SIMCA. 95% confidence intervals are presented within parentheses.
