# Supplementary material for: Prediction of Clinical Remission with Adalimumab Therapy in Patients with Ulcerative Colitis by Fourier Transform–Infrared Spectroscopy Coupled with Machine Learning Algorithms
Source: Metabolites. 2023 Dec 19;14(1):2. doi: 10.3390/metabo14010002 (PMC10818421; doi:10.3390/metabo14010002)
Supplement: Supplementary file 1 [file metabolites-14-00002-s001.zip › Table S6.pdf]

Table S6. Comparison of prediction performance of the remission prediction model at 8 and 56 weeks of adalimumab treatment in patients with ulcerative colitis by various ML algorithms in internal and external validation process.

Week 8	Development model (10F-cross validation)										Validation model				
	Train					Test									
Methods	Accuracy	Precision	Recall	F1_score	ROC-AUC	Accuracy	Precision	Recall	F1_score	ROC-AUC	Accuracy	Precision	Recall	F1_score	ROC-AUC
KNN	0.99 (0.99-1.00)	1.00	0.96 (0.94-0.98)	0.98 (0.97-0.99)	0.98 (0.97-0.99)	0.96 (0.91-1.01)	0.98 (0.93-1.03)	0.91 (0.79-1.03)	0.93 (0.86-1.01)	0.95 (0.89-1.01)	0.454	0.450	0.450	0.450	0.380
DT	0.94 (0.92-0.97)	0.87 (0.79-0.95)	0.91 (0.88-0.93)	0.89 (0.84-0.93)	0.93 (0.91-0.95)	0.9 (0.84-0.95)	0.86 (0.73-1.00)	0.75 (0.55-0.95)	0.75 (0.61-0.89)	0.85 (0.76-0.94)	0.424	0.560	0.420	0.410	0.560
RF	0.96 (0.94-0.97)	0.99 (0.98-1.01)	0.83 (0.76-0.90)	0.90 (0.86-0.94)	0.91 (0.88-0.95)	0.85 (0.79-0.91)	0.95 (0.84-1.06)	0.45 (0.28-0.62)	0.58 (0.44-0.73)	0.72 (0.64-0.81)	0.454	0.420	0.450	0.430	0.460
linear SVM	1.00	1.00	1.00	1.00	1.00	1.00	1.00	1.00	1.00	1.00	0.636	0.640	0.640	0.620	0.750
rbf SVM	1.00	1.00	1.00	1.00	1.00	1.00	1.00	1.00	1.00	1.00	0.545	0.520	0.550	0.530	0.440

Week 56	Development model (10F-cross validation)										Validation model				
	Train					Test									
Methods	Accuracy	Precision	Recall	F1_score	ROC-AUC	Accuracy	Precision	Recall	F1_score	ROC-AUC	Accuracy	Precision	Recall	F1_score	ROC-AUC
LR	1.00	1.00	1.00	1.00	1.00	0.97 (0.95-1.00)	0.99 (0.97-1.01)	0.96 (0.92-1.01)	0.97 (0.95-1.00)	0.97 (0.95-1.00)	0.606	0.580	0.610	0.560	0.580
KNN	0.99 (0.98-0.99)	1.00 (0.99-1.00)	0.98 (0.97-0.99)	0.99 (0.98-0.99)	0.99 (0.98-0.99)	0.94 (0.89-0.98)	0.97 (0.91-1.02)	0.9 (0.8-1.00)	0.92 (0.86-0.99)	0.93 (0.87-0.98)	0.667	0.650	0.670	0.660	0.590
RF	0.99 (0.98-0.99)	0.99 (0.99-1.00)	0.97 (0.95-0.98)	0.98 (0.98-0.99)	0.98 (0.98-0.99)	0.9 (0.84-0.96)	0.98 (0.91-1.04)	0.83 (0.71-0.95)	0.88 (0.81-0.95)	0.90 (0.85-0.96)	0.545	0.490	0.550	0.500	0.310
linear SVM	1.00	1.00	1.00	1.00	1.00	0.97 (0.95-1.00)	0.99 (0.97-1.01)	0.96 (0.92-1.01)	0.97 (0.95-1.00)	0.97 (0.95-1.00)	0.636	0.610	0.640	0.600	0.640
rbf SVM	1.00	1.00	1.00	1.00	1.00	0.98 (0.93-1.03)	1.00	0.96 (0.88-1.05)	0.98 (0.92-1.03)	0.98 (0.94-1.02)	0.545	0.530	0.550	0.490	0.520

95% confidence interval was indicated within parenthesis with the parameter values in development model. DT, decision tree; KNN, K-nearest neighbors; LR, logistic regression; rbf, radial basis function; RF, random forest; ROC-AUC, receiver operating characteristic-area under the curve; SVM, support vector machine; UC, ulcerative colitis. LR, KNN, DT, RF, and SVM were performed by SciKit-Learn software and parameters were selected by the function of "GridSearchCV" in SciKit-Learn software.
